# Mechanisms of Resistance to Rituximab Used for the Treatment of Autoimmune Blistering Diseases

**DOI:** 10.3390/life14101223

**Published:** 2024-09-25

**Authors:** Liliana Gabriela Popa, Ioana Dumitras, Calin Giurcaneanu, Ovidiu Berghi, Diana Sabina Radaschin, Cristina Iolanda Vivisenco, Marius Nicolae Popescu, Cristina Beiu

**Affiliations:** 1 Department of Dermatology, Carol Davila University of Medicine and Pharmacy, 37 Dionisie Lupu Street, District 1, 020021 Bucharest, Romania; 2 Department of Dermatology, Elias Emergency University Hospital, 17 Marasti Bd., District 1, 011461 Bucharest, Romania; 3 Department of Allergy and Clinical Immunology, Colentina Clinical Hospital, 19-21 Stefan cel Mare Bd., District 2, 020125 Bucharest, Romania; 4 Department of Dermatology, Dunarea de Jos University of Medicine and Pharmacy, 25 Otelarilor Bd., 800008 Galati, Romania; 5 Department of Paediatrics, Carol Davila University of Medicine and Pharmacy, 37 Dionisie Lupu Street, District 1, 020021 Bucharest, Romania; 6 Department of Pediatrics, Grigore Alexandrescu Clinical Emergency Hospital for Children, 30-32 Iancu de Hunedoara Road, 011743 Bucharest, Romania; 7 Department of Physical and Rehabilitation Medicine, Carol Davila University of Medicine and Pharmacy, 37 Dionisie Lupu Street, District 1, 020021 Bucharest, Romania; 8 Department of Physical and Rehabilitation Medicine, Dermatology Department, Elias Emergency University Hospital, 17 Marasti Bd., District 1, 011461 Bucharest, Romania

**Keywords:** autoimmune blistering diseases, pemphigus, rituximab, mechanism of action, resistance

## Abstract

Autoimmune blistering diseases represent a group of chronic severe, disabling, and potentially fatal disorders of the skin and/or mucous membranes, primarily mediated by pathogenic auto-antibodies. Despite their rarity, these diseases are associated with significant morbidity and mortality and profound negative impact on the patient’s quality of life and impose a considerable economic burden. Rituximab, an anti-CD-20 monoclonal antibody, represents the first line of therapy for pemphigus, regardless of severity and a valuable off-label therapeutic alternative for subepidermal autoimmune blistering diseases as it ensures high rates of rapid, long-lasting complete remission. Nevertheless, disease recurrence is the rule, all patients requiring maintenance therapy with rituximab eventually. While innate resistance to rituximab in pemphigus patients is exceptional, acquired resistance is frequent and may develop even in patients with initial complete response to rituximab, representing a real challenge for physicians. We discuss the various resistance mechanisms and their complex interplay, as well as the numerous therapeutic alternatives that may be used to circumvent rituximab resistance. As no therapeutic measure is universally efficient, individualization of rituximab treatment regimen and tailored adjuvant therapies in refractory autoimmune blistering diseases are mandatory.

## 1. Introduction

Autoimmune blistering diseases are a group of chronic severe, disabling, and potentially fatal disorders of the skin and/or mucous, primarily mediated by pathogenic auto-antibodies. These diseases include pemphigus, bullous pemphigoid (BP), mucous membrane pemphigoid (MMP), linear immunoglobulin (Ig) A dermatosis (LABD), dermatitis herpetiformis (DH), and epidermolysis bullosa acquisita (EBA) [1]. Pemphigus is characterized by intraepidermal acantholysis triggered by autoantibodies targeting desmogleins (Dsg 1 and 3 in pemphigus vulgaris and Dsg 1 in pemphigus foliaceus), which are crucial for epithelial cell adhesion [1,2]. In contrast, pemphigoid-type and IgA-mediated bullous diseases are characterized by subepidermal blistering, induced by autoantibodies directed against basement membrane zone molecules. In BP, autoantibodies target the 230 kilodalton (KDa) type XVII collagen, referred to as bullous pemphigoid antigen (BPAg)1 and BPAg2, a 180 KDa hemidesmosomal protein [3]. In MMP, autoantibodies are directed against multiple antigens, including BPAg1, BPAg2, laminin 5, α6β4 integrin, and type VII collagen [3]. IgA1 autoantibodies that mediate LABD usually target two extracellular components of BPAg2 with a molecular weight of 97 and 120 KDa, respectively, and are only rarely directed against type VII collagen, laminin 5, or laminin γ1 [4]. DH arises in genetically predisposed individuals suffering from gluten sensitivity and is characterized by the production of anti-epidermal transglutaminase IgA autoantibodies. [5]. EBA is caused by anti-type-VII collagen antibodies, which impair the adhesion of the basement membrane to the dermis [1,6].

The reported incidence of pemfigus vulgaris (PV), the most common form of pemphigus ranges between 0.1–5/100,000/year [1]. While the onset of pempigus usually takes place during the fifth and sixth decades of life [2], BP and MMP primarily affect the elderly, typically occurring after the age of 60 [3]. BP has an estimated incidence of 0.4–2.2 cases/100,000/year, being the most common autoimmune blistering disease, especially in the elderly, reaching an incidence of 18.9 cases/100,000/year in individuals older than 80 [3]. MMP is rarer, with an incidence of approximately 0.2 cases/100,000/year [3]. The incidence of DH is estimated at 0.4–3.5 cases/100,000/year [5]. LABD and EBA are very uncommon disorders, with incidences of 0.05–0.23 cases/100,000/year [4] and 0.026 cases/100,000/year, respectively [6].

Despite their rarity, these diseases are associated with significant morbidity and mortality, profoundly impacting patients’ quality of life. They also impose a considerable economic burden, as they usually require long-term treatment and frequent, thorough monitoring due to the high risk of complications from both the disease and its aggressive treatment. Even with modern therapies, pemphigus still carries a mortality rate of 5–10%, with a substantial proportion attributable to treatment complications [7].

The mainstay of therapy for these conditions has traditionally been represented by systemic corticosteroids (CS) combined with steroid-sparing agents, especially conventional immunosuppresants. Although these treatments have significantly reduced mortality, the adverse effects of long-term, high-dose systemic corticotherapy and immunosuppressive agents are redoubtable. Prolonged corticotherapy may lead to cardiovascular complications (arterial hypertension, ischemic heart disease, cardiac failure), endocrine and metabolic adverse effects (diabetes mellitus, dyslipidemia, obesity, iatrogen Cushing’s syndrome, growth suppression), gastrointestinal side effects (gastritis or gastric/duodenal ulcer), musculoskeletal disorders (myopathy, osteoporosis, aseptic osteonecrosis), ocular complications (cataract, glaucoma), neuropsychiatric disorders (from anxiety and irritability to frank psychosis), increased risk of infection, and cutaneous side effects (skin atrophy, purpura, striae, impaired wound healing, acne, hirsutism) [7,8]. Corticosteroid-sparing immunosuppressants like azathioprine, mycophenolate mofetil, cyclophosphamide, and cyclosporine further increase the patients’ risk of infection, may cause myelosuppression, gastrointestinal complications, impaired renal and liver function, and increased risk of malignancy [7,8]. Additionally, severe forms of autoimmune blistering diseases refractory to conventional anti-inflammatory and immunosuppressive therapies are not uncommon and often necessitate alternative treatment strategies, like plasmapheresis or high dose intravenous immunoglobulins. In recent decades, the use of anti-CD20 monoclonal antibodies in patients with severe, unresponsive autoimmune blistering diseases has proven highly effective, leading to the recommendation of rituximab as first line treatment not only for moderate or severe, but also for mild cases of pemphigus, according to the European Academy of Dermatology and venereology (EADV) pemphigus treatment guidelines [8]. Subepidermal autoimmune blistering diseases also greatly benefit from the off-label use of rituximab, which achieves high rates of long-lasting complete clinical remission, as well as serological remission in patients who fail conventional treatment [9,10,11,12].

## 2. Mechanism of Action of Rituximab in Autoimmune Blistering Diseases

Rituximab is a chimeric monoclonal antibody composed of human IgG1 immunoglobulin constant regions and murine variable regions, specifically targeting CD20, a transmembrane protein structurally similar to the β subunit of the high-affinity immunoglobulin E receptor (FcεR) I, important for the differentiation, growth, and activation of B lymphocytes [13]. Originally intended for treating B-cell-hematologic malignancies, rituximab has proven effective in numerous autoimmune diseases given its complex mechanism of action.

**Figure 1:**
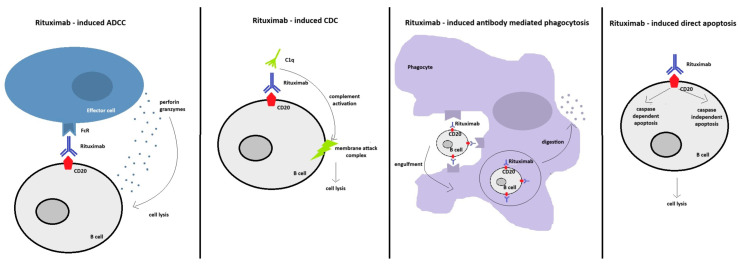
Main mechanisms of CD20+ B cells depletion induced by rituximab. Abbreviations: ADCC—antibody-dependent cellular cytotoxicity (ADCC); CDC—complement-dependent cytotoxicity; C1q—complement fragment 1q.

The primary effect of rituximab is the rapid and long-lasting depletion of CD20+ B cells, including peripheral mature B cells, as well as bone marrow immature B cells, autoantigen-activated marginal zone and follicular B cells. This depletion is achieved principally, but not exclusively, through antibody-dependent cellular cytotoxicity (ADCC). Direct induction of apoptosis, antibody-mediated phagocytosis, and complement-dependent cytotoxicity (CDC) contribute to CD20+ B cells death (Figure 1) [14]. Autoreactive memory B-cells (MBCs) are particularly affected by rituximab, which accounts for its prompt and sustained therapeutic effect in autoantibody-mediated autoimmune conditions [14,15,16,17,18]. Notably, rituximab does not impact CD20− pro-B cells, allowing repopulation of peripheric blood with B cells within 6–12 months [16]. Long-lived plasma cells (LLPCs), which are CD20−, are also spared, explaining the insignificant variation in total serum antibody levels and anti-infectious antibody levels following treatment with rituximab [16]. Upon B-cell repopulation, defined by Albers et al. as the presence of ≥5 CD19+ cells/μL [19], apart from the markedly increased naive/MBC ratio, the expansion of circulating regulatory B-cells (Bregs) occurs [17]. Most of these Bregs are transitional interleukin 10 (IL-10) producing B cells, able to reduce the antigen-presenting potential of dendritic cells, as well as CD4+ T cells responses [17]. This contributes to the maintenance of immune tolerance and disease remission even after reemergence of mature B cells [20]. While the population of IL-10 producing B cells increases following treatment with rituximab or IVIg, this does not occur following corticotherapy and treatment with conventional immunosuppressive agents [21].

Several other mechanisms confer rituximab its curative valences. Although it does not influence the total number and function of peripheral CD4+ and CD8+ T cells [22,23], rituximab induces a swift and prolonged decline in autoreactive CD4+ T cells owing to the loss of the stimulation exerted by autoreactive B cells acting as antigen-presenting cells [24]. Another possible explanation is the depletion of CD20+ T helper (Th) 17 cells, as demonstrated in patients with rheumatoid arthritis [25]. The number of autoreactive T cells correlates with the serum levels of autoantibodies and with the clinical activity of the disease [23,24]. Moreover, circulatory regulatory T cells (Treg) numbers are reduced in PV patients, contributing to the overactivity of autoreactive B cells [26,27]. Unlike other autoimmune diseases, in PV they decrease even more after rituximab administration, only to be detected in higher numbers in lesional skin [28], suggesting an increased Tregs skin homing meant to contain the cutaneous autoimmune process [29]. This rituximab-specific response further aids in disease control.

**Figure 2:**
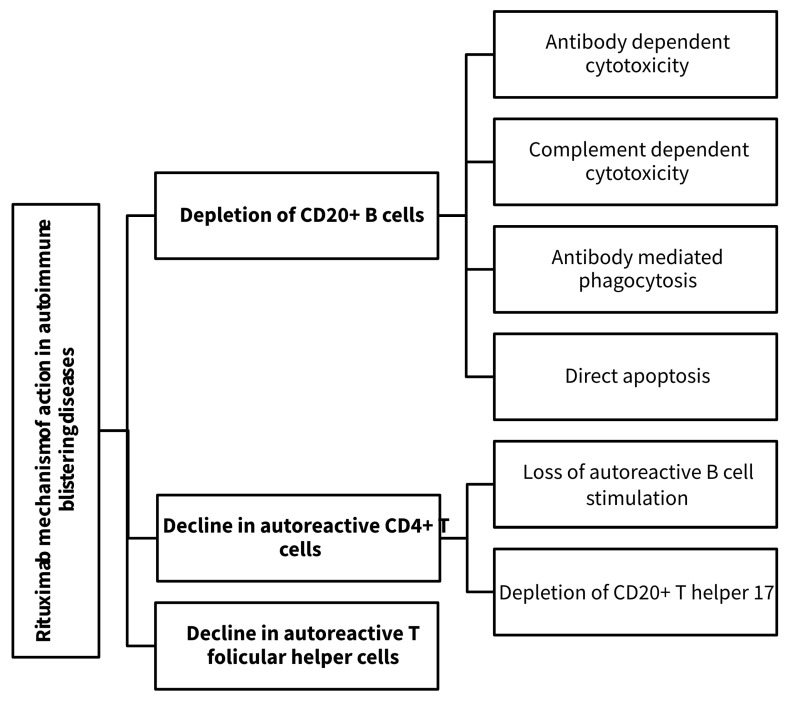
Rituximab’s mechanisms of action in autoimmune blistering diseases.

Rituximab exerts other specific effects, as depicted in Figure 2. It causes a substantial decrease in the number of autoreactive T follicular helper (Tfh) cells in peripheral blood, although the circulating Tfh cells total number is not modified. The drop in autoreactive Tfh cells numbers is associated with a considerable decrease in serum IL-21 levels [30]. The latter correlates with B-cell depletion given the critical role of IL-21 in B-cell maturation, particularly MBC generation [30]. On the other hand, as demonstrated by Baumjohann et al., to maintain their phenotype, Tfh cells require constant antigenic stimulation by B cells. Therefore, the impact is bidirectional, as the depletion of autoreactive B cells hampers autoreactive Tfh cells development and maintenance [31].

B cells repopulate the peripheral blood 6–12 months after the administration of rituximab [16]. However, these are naïve B cells. B cell maturation is hindered for a much longer period of time, delaying the reappearance of MBCs [32]. The naive/MBC ratio is markedly increased, explaining the lack of increase in serum autoantibody levels after B-cell repopulation [16]. Furthermore, the newly generated B cells display rearrangement of their Ig repertoire, converting from oligoclonal to polyclonal [17]. Despite this, persistently high autoantibody levels have been reported in 16–40% of patients in complete remission [33,34]. These are most likely non-pathogenic antibodies, targeting different epitopes [17]. Thus, B cell repopulation of the peripheral blood is usually not associated with disease recurrence [32].

The serum level of B cell-activating factor (BAFF), a key factor in B-cell maturation, is high while MBCs are absent from peripheral blood and greatly decreases upon B-cell recovery, signaling the risk of disease recurrence [35]. Rituximab specifically decreases BAFF-R mRNA in non-autoreactive, as well as in reemerging autoreactive B cells, an effect that contributes to sustained remissions despite B cell repopulation [36].

## 3. Disease Relapse after Rituximab Administration

Disease relapse occurs in virtually all patients with autoimmune blistering diseases after a variable period of time, usually 6 to 24 months following administration of rituximab [19,37]. This is due to several interconnected processes. 

Autoreactive MBCs can persist in the spleen and lymph nodes given the intense survival signals in these areas [15]. This incomplete MBC depletion does not always lead to an early relapse, as these cells may remain dormant in lymphoid organs for years, even decades [15]. Persistence of autoreactive CD4+ Th cells is also possible. 

The other main mechanism underlying disease recurrence is the emergence of new lineages of autoantigen-specific B cells. Autoantibody-producing LLPCs may also induce disease recurrence [16].

Given these factors, all patients need maintenance treatment eventually, yet the optimal regimen is to be established. The current EADV guidelines for the management of pemphigus recommend maintenance treatment with rituximab administered 6 months after the initial cycle in doses ranging from 500 mg to 1 g in patients who achieved complete remission, especially patients with severe PV at initial presentation and/or patients with high anti-Dsg antibodies levels at month 3 and a full cycle (two infusions of 1 g two weeks apart) in patients without complete remission. Subsequently, a dose of 500 mg of rituximab every 6 months is recommended [8]. However, some experts argue that additional doses of rituximab should be administered only in patients with incomplete response or disease recurrence. We also endorse this approach, as the majority of our patients have experienced long-lasting complete remission, ranging from 18 months to 13 years, without any specific treatment following rituximab administration. 

In the absence of large, randomized controlled trials, data on the use of rituximab in other autoimmune blistering diseases stems from case reports and case series. Recurrences after the initial rituximab cycle occurred after a mean interval of 10 months and were successfully treated with additional doses of rituximab [9,12]. 

In our view, frequent administration of rituximab, a profoundly immunosuppressive therapy, in patients with sustained complete remission is unnecessary and unjustified. We believe maintenance therapy should be individualized. Ideally, subsequent administrations of rituximab should be prompted by the detection of biomarkers that signal an imminent recurrence of the autoimmune blistering disease. Such biomarkers are under investigation and include circulating CD19+ B cell counts, the speed of B cell depletion and repopulation [28], CD4+ T cell counts, pathogenic anti-Dsg autoantibody serum levels, BAFF serum level, as well as several genetic markers predicting response to rituximab [19,38,39].

## 4. Mechanisms of Resistance to Rituximab and Strategies to Overpass It

Innate resistance to rituximab in patients with autoimmune blistering diseases is exceptional and is usually due to low CD20 expression or accelerated drug clearance [13]. Acquired resistance, on the other hand, is frequent and can develop even in patients with initial complete response to rituximab. Apart from the evading mechanisms already discussed, a series of additional mechanisms may be involved in the development of rituximab resistance, such as CD20 downregulation, especially on MBCs [40]; impaired rituximab–CD20 binding due to the release of human anti-chimeric antibodies (HACAs) to rituximab [41]; and CD20 alterations like CD20 alternative transcript (D393–CD20) [42] or lipid raft signaling biochemical changes [43]. Certain comorbidities, previous treatments and genetic variations and mutations also impact the immunological effects of rituximab [43].

1. **Interference with rituximab’s mechanisms of action**

CD46, CD59, and CD55 are membrane proteins that inhibit complement activation and the formation of the membrane attack complex. B cells that overexpress these complement activation regulators are resistant to rituximab because they are not susceptible to CDC. This might represent a result of selective pressure due to previous exposure to rituximab [43]. The purine analog fludarabine seems to act synergically with rituximab, an effect explained by its ability to decrease CD55 activity [44], making it a useful adjuvant therapy in such cases of resistance to rituximab. However, as the role of complement activation regulators in the protection of normal cells against CDC is crucial, aggressive downregulation of these proteins would be unquestionably detrimental. 

Rituximab-induced CDC may also be hindered by the consumption of complement proteins. Klepfish et al. proved that coadministration of fresh frozen plasma with rituximab successfully counteracts this deficiency and restores rituximab’s efficacy [45].

Nevertheless, there is a very fine line between benefit and harm with such interferences. As previously discussed, rituximab exerts its destructive effect on B cells mainly through ADCC. The ability of natural killer (NK) cells to perform ADCC is impeded by C3b, therefore complement depletion favors this process [46]. Furthermore, upregulation of human leukocyte antigen (HLA) I on B cells can render them resistant to NK-cell-mediated ADCC [47]. 

ADCC is also hindered by a defective binding of rituximab to its target and by conformational changes in the CD20/rituximab complex [43]. In some patients, FcR polymorphisms may be the cause of rituximab failure to induce ADCC. Alteration of the lipid rafts of B cells membranes in patients receiving statins was demonstrated to induce in vitro resistance to ADCC [48]. Some authors also point to vitamin D deficiency as potentially involved in rituximab-mediated ADCC resistance [49]. Administering rituximab in combination with granulocyte colony-stimulating factor (G-CSF), granulocyte macrophage colony stimulating factor (GM-CSF), interferon (IFN)-γ, IL-2, IL-12, and IL-15 may help surpass ADCC resistance by increasing the function of NK cells [43]. Most studies have focused on the effects of G-CSF and GM-CSF, which upregulate the expression of neutrophil adhesion molecules [50] and IL-2, which also augments ADCC [51]. The potential of CpG oligonucleotides, bromohydrin pyrophosphate, and Toll-like-receptor 9 (TLR-9) agonists to enhance ADCC is currently under investigation [52,53,54].

Last but not least, after several courses of rituximab, B cells may become resistant to rituximab—induced apoptosis, mainly due to the excessive activation of the nuclear factor—κB (NF-κB) pathway, known to markedly stimulate cellular antiapoptotic mechanisms. This triggers overexpression of the anti-apoptotic proteins belonging to the Bcl-2 family, whereas Bax and Bak, the pro-apoptotic members of the Bcl-2 family proteins, are substantially down-regulated, leading to resistance not only to rituximab but also to chemotherapy [55]. Combination therapy with rituximab and Bcl-2 inhibitors like oblimersen has shown encouraging results in follicular NHL patients, achieving response rates of 60%, even in rituximab—resistant cases [56]. Several other therapeutic agents, such as temsirolimus, bortezomib, and histone deacetylase inhibitors, also sensitize lymphoma cells to rituximab in vitro [57,58]. Moreover, in lymphoma patients, resistance to apoptosis can be overcome by combined treatment with rituximab and cytotoxic agents [43]. Researchers are exploring techniques to increase CD20 expression in order to restore B cells susceptibility to rituximab-induced apoptosis.

2. **Altered CD20 expression**

Apart from the development of resistance to rituximab-induced CDC, ADCC, and direct apoptosis, other factors may impact the treatment’s effectiveness. Among these, the most important is represented by the altered expression of CD20 on the surface of B cells, which may be caused by a variety of processes, such as lipid raft reorganization and perturbed signaling, CD20 internalization upon repeated administration of rituximab, and genetic variations [43]. CD20 gene deletion mutations affecting the C-terminal region impair antibody binding [43]. The exon 2–216 C/T polymorphism of the gene encoding CD20, believed to influence its expression and mRNA stability greatly impacts the response to rituximab in B cell lymphoma patients, C/C homozygotes achieving significantly greater remission rates compared to T/T homozygotes or heterozygotes [59]. In addition, Shimizu et al. explored the epigenetic factors linked to decreased CD20 expression and demonstrated the ability of valproic acid and romidepsin, both histone deacetylase inhibitors to increase CD20 expression in vitro and subsequently amplify rituximab-induced CDC [58].

3. **Removal of CD20-rituximab complexes from the B-cell surface**

Another interesting resistance mechanism is the removal from the B cell surface of CD20-rituximab complexes upon recognition by FcR on macrophages and monocytes. However, this so-called “shaving” of CD20-rituximab complexes can be avoided by adding intravenous immune globulines (IVIGs) to the treatment regimen [60].

4. **Production of antibodies against rituximab**

No large studies have analyzed the production of HACAs in patients receiving rituximab. The available evidence indicates that HACAs are more often detected in patients treated with rituximab for autoimmune diseases, particularly systemic lupus erythematosus, compared to patients with NHL [41]. However, the production of HACAs to rituximab is a rare phenomenon and interestingly occurs late, usually 18 to 44 weeks after the first dose of rituximab [41]. Additionally, in some patients, HACA production proved transient. The infusion protocol and concomitant use of immunosuppressants do not seem to influence HACAs production [41]. The clinical importance of HACAs to rituximab is still a matter of debate, but the risk of hypersensitivity reactions and incomplete control of the disease should not be overlooked.

5. **Genetic factors**

Other genetic alterations influence rituximab pharmacokinetics, efficacy and safety in autoimmune diseases [37]. 

Given the crucial role of FcγR, particularly that of the stimulatory FCγRIIIA in the activity of macrophages and NK cells, the influence of FCGR3A gene polymorphisms on rituximab efficacy has been investigated [61,62,63]. FCγRIIIA displays stronger affinity for IgG, leading to enhanced cytotoxicity and B cell depletion and, implicitly, a superior response to rituximab in carriers of FCGR3A-158V (rs396991; T > G) polymorphism [64,65,66,67], i.e., valine homozygotes (V/V) compared to phenylalanine homozygotes (F/F) and heterozygotes (V/F) [68,69,70,71]. Secondary resistance to rituximab was only reported in the latter two patient cathegories [66]. FCGRA-158 F/F carriers also present a higher risk of hypogammaglobulinaemia [72] and sepsis [73] following rituximab treatment. On the other hand, FCGRA-158 V/V and FCGRA-158 V/F polymorphisms are associated with a higher risk of rituximab-related late-onset neutropaenia [74].

The structure of FcγRIIA, a stimulating receptor expressed by macrophages and monocytes also impacts the receptor’s antibody binding intensity and the clinical response to rituximab. FCGR2A-131 histidine homozygotes (H/H) proved considerably more responsive to rituximab than arginine homozygotes (R/R) or heterozygotes (H/R) [75,76,77]. Moreover, FCGR2A-131 H/H and FCGR2A-131 H/R carriers have an increased risk of anemia, hypogammaglobulinemia and sepsis post-rituximab treatment compared to R/R homozygotes [78,79].

Polymorphisms of genes encoding cytokines essential for the survival, proliferation and maturation of B cells also greatly influence the response to rituximab [37]. Among them, GC/GG genotypes of the IL-6 promoter at position −174 (rs1800795) are associated with a better response to rituximab 6 months after the initial cycle, accompanied by a significant decrease in IL-6 serum levels in rheumatoid arthritis patients [80]. On the contrary, −174 CC homozygotes are much less likely to respond to rituximab and present a much lesser decrease in IL-6 levels 6 months after rituximab administration [80]. 

Daien et al. studied the influence of 9 gene variations and 13 single nucleotide polymorphisms (SNPs) on the response to rituximab in patients diagnosed with rheumatoid arthritis and reported a positive correlation between SNPs at transforming growth factor (TGF)-β1 codon 25 (rs1800471—the G/C genotype) and codon 10 (rs1800470—the C/T genotype) and rituximab efficacy [81]. This is not surprising considering the inhibitory role of TGF-β1 on the activation of B cells and antibody production [81].

The impact of numerous interferon (IFN) type I response genes (IRGs), like LY6E, HERC5, IFI44L, ISG15, MxA, MxB, EPSTI1, RSAD2, and OAS1 on rituximab efficacy in rheumatoid arthritis patients was evaluated in several studies [82,83]. Both the three IRGs cluster (Mx1, ISG15 and OAS1) and the eight IRGs cluster (LY6E, HERC5, IFI44L, ISG15, MxA, MxB, EPSTI1, RSAD2) showed a strong inverse correlation with the clinical response to rituximab, independent of other predictive factors, such as the presence of autoantibodies and the previous use of biologic treatments, disease modifying antirheumatic drugs (DMARDs), and statins [82,83]. Cantaert et al. hypothesized that B cells play a less significant role in the pathogenesis of rheumatoid arthritis in patients with high IFN-α and -β serum levels [84]. This hypothesis has been contradicted by other authors considering that IRG expression does not vary depending on the presence of absence of autoantibodies [85]. Cambridge et al. suggested the possible association of high IFN type I levels with rituximab-insensitive B cells [85]. 

The impact of a series of SNPs for IFN regulatory factor 5 (IRF5) (rs2004640), IRF7 (rs1131665) tyrosine kinase (TYK) 2 (rs2304256, rs280519 and rs12720356), signal transducer and activator of transcription (STAT) 4 (rs7574865), and osteopontin (SPP1) (rs11439060 and rs9138) on rituximab effectiveness have also been assessed [86]. SPP1 rs9138 A/A and IRF5 rs2004640 G/T or G/G genotypes strongly correlated with a good response to rituximab, independent of the presence or absence of autoantibodies [86]

The G/T polymorphism of the IL2/IL21 region (rs6822844) also affects rituximab efficacy in patients with systemic lupus erythematosus, G/G homozygotes responding better to rituximab [87]. This correlation did not apply to patients with other autoimmune diseases [87]. 

Genetic variations of another key factor for B cell survival and maturation, BAFF, have been analyzed, leading to the conclusion that in patients with rheumatoid arthritis, homozygous C/C genotypes of BAFF promoter at position 871 (rs9514828) are associated with a superior response to rituximab than T/T genotypes [88]. Additionally, patients with the TTTT BAFF promoter haplotype (871 C > T, 2704 T > C, 2841 T > C and 2701 T > A) show a better response to rituximab [89]. Contrarily, C/C homozygotes for BAFF-2704 and carriers of SNP rs3759467 in the 5′ regulatory region of BAFF gene who received rituximab for the treatment of anti-neutrophil cytoplasmic antibody (ANCA) vasculitis were more frequently resistant to rituximab [90].

In addition, a positive correlation has been observed in seropositive rheumatoid arthritis patients between rituximab efficacy and the presence of allele 2 of HS1.2A enhancer of the 3′ regulatory region of the heavy Ig chain, explained by its role in B cell maturation and autoantibody production [91].

The intensity of rituximab-induced CDC is positively correlated with the serum levels of the complement fragment C1q [92]. However, G/A polymorphisms of C1QA-276 have a contradicting effect, the A/A genotype being associated with lower serum C1q levels [93], but at the same time with greater remission rates and a more durable response in patients with B cell lymphomas compared to A/G and G/G genotypes [92,94].

6. **Previous treatments**

Previous treatments received by the patient may also influence rituximab efficacy. In a large observational study, Chatzidionysiou et al. analyzed the data provided by 10 countries registries regarding 2019 rheumatoid arthritis patients treated with rituximab and concluded that the most important predictors of response to rituximab were seropositivity for rheumatoid factor or anti cyclic citrullinated peptide antibodies, not having previously received biologic treatment or having undergone treatment with only one biological agent before rituximab [95]. Resistance to rituximab was more frequently observed among patients who had been unsuccessfully treated with more than one anti-tumor necrosis factor (TNF) α agent [95]. These findings are supported by the results of other studies [77,96]. Moreover, previous studies showed co-administration of classical DMARDs leads to a more sustained clinical benefit after the first cycle of rituximab in patients with rheumatoid arthritis [95]. Although this is also true for patients with autoimmune blistering diseases, the concomitant administration of conventional immunosuppressive agents is usually avoided due to the greater risk of adverse effects, especially infections [97].

7. **Rituximab pharmacokinetics**

As higher serum concentrations of rituximab were detected in responders vs. non-responders in B cell non-Hodgkin lymphoma (NHL) patients [43], it is only intuitive that higher doses or more frequent administrations could benefit non-responders with autoimmune blistering diseases. Serum concentrations of rituximab depend on multiple factors, among which are genetic factors, disease type and tumor burden [43]. FcγRIII gene polymorphism is one such factor, shown to influence rituximab concentrations and efficacy [98]. Dayde et al. demonstrated an inverse correlation between rituximab serum concentrations and tumor burden in NHL patients as rituximab readily infiltrated highly vascularized tumors given the abundance and availability of antigens [99]. While rituximab’s highly variable pharmacokinetics most probably plays a key role in resistance, the minimum effective rituximab serum concentration remains unclear. Although some authors support the use of higher doses of rituximab (e.g., 1 g/m^2^) in non-responders [100], no large studies have addressed this issue so far.

Interestingly, intralesional rituximab is a valuable therapeutic option even in patients refractory to intravenous rituximab. Vinaj et al. reported marked improvement of the oral lesions in three PV patients refractory to conventional immunosuppressants and intravenous rituximab [101]. Yuan et al. detected the accumulation of Dsg-3 and Dsg-1 specific B-cells in pemphigus cutaneous lesions and postulated that the skin acts as a tertiary lymphoid organ, with infiltrating auto-reactive B-cells releasing anti-Dsg autoantibodies due to an intense cross-talk between B cells and IL-21- and IL-17A-producing CD4+ T cells. Thus, lesions resistant to intravenous rituximab may remain susceptible to the intralesional administration of the drug [102]. Table 1 summarizes the mechanisms of resistance to rituximab. 

### Other Strategies to Overpass Rituximab Resistance

An alternative approach in rituximab resistant cases is the use of second and third generation humanized or completely human anti-CD20 monoclonal antibodies. These possess a series of advantages over rituximab, including significantly lower immunogenicity and superior efficacy due to higher affinity for FcR, enhanced ADCC (ocrelizumab, GA-101), enhanced ADCC, and direct apoptosis (obinutuzumab), greater binding avidity, improved CDC, and slower dissociation rates (veltuzumab), more potent CDC due to the stronger binding to CD20 at a more proximal site to the cell membrane and a slower dissociation rate, as well as resistance to the effects of complement-regulatory molecules (ofatumumab) [43,104,105,106]. As evidence of their ability to surmount rituximab resistance mechanisms stems from a limited number of case reports, further studies are needed to assess their efficacy in resistant cases of autoimmune blistering diseases.

BAFF-targeting therapies such as belimumab and atacicept have also been successfully used in PV patients resistant to rituximab [103].

Treatment with ibrutinib is another appealing strategy in rituximab-resistant patients with autoimmune blistering diseases. The Bruton kinase (BTK) inhibitor has proven highly effective not only in patients with B-cell lymphomas, but also in pemphigus. BTK is principally expressed on B-cells, except plasma cells and its activation leads to stimulation of p38MAPK, NFkB and MEK/ERK pathways, resulting in B-cell proliferation and maturation, autoantibody production and Ig class switching [103,107,108], which implicitly favor Tfh cells differentiation. Furthermore, topical p38MAPK inhibitors may prove useful and safe adjuvants in refractory patients [103].

Janus kinases (JAK) 1 and 3 inhibitors (tofacinib) may also be used as adjuvants in refractory PV, even as a topical treatment as they suppress the activation of Dsg-specific Tfh cells and their influence on B cells phenotype and function [103,109].

STAT 3 inhibitors (rapamicin, mTOR inhibitors such as sirolimus, or STAT3 inhibitor XVIII) were shown to be effective in PV in animal studies by increasing Dsg3 expression [110,111].

Effects of anti CD19+ monoclonal antibodies (inebilizumab), which also act on plasma cells are studied in PV given that the production of anti-Dsg autoantibodies by LLPCs represents one of the mechanisms of rituximab resistance. CD19-directed CAR-T-cell therapy and Dsg3-CAAR T cells are also being studied in PV, showing very promising preliminary results [112,113].

Considering the significant decrease of Dsg-specific Tregs observed in PV patients, the benefit of autologous polyclonal Tregs infusion in PV and PF patients is under investigation in a phase-1 open-label multicenter trial (NCT03239470) [114]. 

## 5. Conclusions

The therapeutic landscape of autoimmune bullous diseases has dramatically changed with the introduction of anti-CD20 monoclonal antibodies. Nowadays, rituximab represents the first line of therapy for pemphigus, regardless of severity, as it ensures high rates of rapid, long-lasting complete remission, often without the need for systemic corticotherapy or conventional immunosuppressive therapy, while maintaining a favorable safety profile. Rituximab is also a valuable off-label therapeutic alternative in subepidermal autoimmune blistering diseases refractory to standard anti-inflammatory and immunosuppressive treatment. Nevertheless, disease recurrence is the rule, all patients requiring maintenance therapy with rituximab eventually. In order to avoid unnecessary supplementary doses, inherent side effects and additional costs, there is an urgent need to identify and validate biomarkers predictive of imminent disease recurrence that would allow the implementation of optimal, personalized therapeutic regimens.

Rituximab resistance in patients with severe, recalcitrant autoimmune blistering diseases remains a significant challenge for the physician. Despite the numerous interesting therapeutic alternatives that offer theoretical promise of circumventing rituximab resistance, clinical studies are warranted in order to prove their potential. The large array of resistance mechanisms and their complex interplay suggest that no single therapeutic approach will be universally effective. Therefore, individualized rituximab treatment regimen and tailored adjuvant therapies for refractory cases are mandatory.

## Figures and Tables

**Table 1:** Mechanisms of resistance to rituximab and strategies to overpass it.

Cathegory	Mechanism	Cause	Premise	Strategies to Circumvent Resistance
**Interference with rituximab’s mechanisms of action**	Impaired CDC	Overexpression of complement activation regulators CD46, CD59 and CD55 on the surface of B cells [43]	Selective pressure due to prior exposure to rituximab	Combination therapy with fludarabine, which decreases CD55 activity [44]
		Consumption of complement proteins	Consumption during CDC	Coadministration of fresh frozen plasma [45]
	Impaired ADCC	Defectuous binding of rituximab to its target Conformational changes in the CD20/rituximab complex [43]	FcR polymorphisms Alteration of the lipid rafts of B cells membranes in patients recieving statins [48] Vitamin D deficiency [49]	Combination therapy with G-CSF, GM-CSF, IFN-γ, IL-2, IL-12, and IL-15, which upregulate the expression of neutrophil adhesion molecules [50] and increase NK cells activity [43] CpG oligonucleotides, bromohydrin pyrophosphate, and TLR-9 agonists seem to enhance ADCC and are currently investigated [52,53,54]
	Impaired direct apoptosis	Excessive activation of NF-κB pathway	Overexpression of the anti-apoptotic proteins and downregulartion of pro-apoptotic proteins [55]	Combination therapy with Bcl-2 inhibitors like oblimersen [56], temsirolimus, bortezomib, and histone deacetylase inhibitors that sensitize lymphoma cells to rituximab in vitro [57,58] or cytotoxic agents in lymphoma patients [43]
**Altered CD20 expression** [43]	Lipid raft reorganization	Perturbed signaling	Biochemical changes	Valproic acid and romidepsin, both histone deacetylase inhibitors seem to increase CD20 expression in vitro [58]
	CD20 internalization	Perturbed signaling	Repeated administration of rituximab	
	Genetic alterations	CD20 gene deletion mutations affecting the C-terminal region, impairing antibody binding [43]	Altered CD20 expression and mRNA stability	
		−216 C/T polymorphism in exon 2 of CD20 gene [59]		
	Epigenetic factors [58]	Decreased CD20 expression	Altered binding of rituximab to CD20	
**Removal of CD20-rituximab complexes from the B-cell surface**	“Shaving” of CD20-rituximab complexes	Removal of rituximab-CD20 complexes upon recognition by FcR on macrophages and monocytes	Saturation of the phagocytic system by previously cleared rituximab opsonized cells and divertion of the rest of rituximab-opsonized cells to different pathways that lead to removal of CD20-rituximab complexes [60]	Combination therapy with IVIGs [60]
**Rapid rituximab clearance**	Production of anti-chimeric antibodies to rituximab [41]	Immune response against rituximab’s murine components	Neutralizing antibodies block rituximab’s active site	Concomitant administration of conventional immunosuppressive agents
**Genetic factors**	FCGR3A-158 polymorphisms [66]	Phenylalanine homozygotes (F/F) Phenylalanine/valine heterozygotes (V/F) [68,69,70,71]	Reduced cytotoxicity and B cell depletion	
	FCGR2A-131 polymorphisms [75,76,77]	Arginine homozygotes (R/R) Histidine/arginine heterozygotes (H/R)	Lower antibody binding intensity	
	-174 IL-6 promoter polimorphisms [80]	CC homozygotes	Lesser decrease in IL-6 levels and poorer response to rituximab	
	IRGs [82,83]	Three IRGs cluster (Mx1, ISG15 and OAS1) Eight IRGs cluster (*LY6E*, *HERC5*, *IFI44L*, *ISG15*, *MxA*, *MxB*, *EPSTI1*, *RSAD2*)	B cells might play a less significant role in the pathogenesis of rheumatoid arthritis in patients with high IFN-α and -β serum levels [84] Possible association of high IFN type I levels with rituximab-insensitive B cells [85].	
	BAFF polymorphisms	T/T genotypes of BAFF promoter at position 871 [88] C/C homozygotes for BAFF-2704 [90] Carriers of SNP rs3759467 in the 5′ regulatory region of BAFF [90]	BAFF is a key factor for B cell survival and maturation.	The use of BAFF targeting therapies such as belimumab and atacicept [103]
	C1QA-276 polymorphisms	A/G and G/G genotypes [92,94]	The intensity of rituximab-induced CDC is positively correlated with C1q serum levels [92].	Coadministration of fresh frozen plasma [45]
**Previous treatments**	Treatment with ≥2 biological agents prior to rituximab [95]	Influence on the biologic effect, immunogenicity of rituximab and drug clearance	Immunologic consequencies of prior biologic therapies	Use of rituximab as first-line biologic treatnent
**Rituximab phamacokinetics**	Lower serum concentrations [43]	Genetic factors Disease type Tumor burden [43]	Rituximab readily infiltrates highly vascularized tumors given the abundance. and availability of antigens [99].	Administering higher doses of rituximab in non-responders [100] Using intralesional rituximab [101]

Abbreviations: CDC—complement-dependent cytotoxicity; ADCC—antibody dependent cytotoxicity; FcR—Fc receptor; G-CSF—granulocyte-colony-stimulating factor; GM-CSF—granulocyte-macrophage-colony-stimulating factor; IFN-γ—interferon-γ; IL—interleukin; NK cells—natural killer cells; TLR-9—toll like receptor-9; IVIGs—intravenous immunoglobulins; IRGs—interferon type I response genes; BAFF—B cell-activating factor; SNP—single nucleotide polymorphism; C1q—complement fragment 1q.

## Data Availability

No new data were created or analyzed in this study. Data sharing is not applicable to this article.
